# Structural characterization and Hirshfeld surface analysis of 2-iodo-4-(penta­fluoro-λ^6^-sulfan­yl)benzo­nitrile

**DOI:** 10.1107/S2056989020000365

**Published:** 2020-01-17

**Authors:** Jean C. González Espiet, Juan A. Cintrón Cruz, Dalice M. Piñero Cruz

**Affiliations:** a Department of Chemistry, University of Puerto Rico-Rio Piedras Campus, PO Box 23346, San Juan, 00931-3346, Puerto Rico; b Department of Chemistry and the Molecular Sciences Research Center, University of Puerto Rico-Rio Piedras Campus, PO Box 23346, San Juan, 00931-3346, Puerto Rico

**Keywords:** substituted arenes, penta­fluoro­thio, functionalized aromatic rings, organometallic synthesis, crystal structure

## Abstract

The title compound, C_7_H_3_F_5_INS, a penta­fluoro­sulfanyl (SF_5_) containing arene, was synthesized from 4-(penta­fluoro­sulfan­yl)benzo­nitrile and lithium tetra­methyl­piperidide following a variation to the standard approach, which features simple and mild conditions that allow direct access to tri-substituted SF_5_ inter­mediates that have not been demonstrated using previous methods. The mol­ecule displays a planar geometry with the benzene ring in the same plane as its three substituents. It lies on a mirror plane perpendicular to [010] with the iodo, cyano, and the sulfur and axial fluorine atoms of the penta­fluoro­sulfanyl substituent in the plane of the mol­ecule. The equatorial F atoms have symmetry-related counterparts generated by the mirror plane. The penta­fluoro­sulfanyl group exhibits a staggered fashion relative to the ring and the two hydrogen atoms *ortho* to the substituent. S—F bond lengths of the penta­fluoro­sulfanyl group are unequal: the equatorial bond facing the iodo moiety has a longer distance [1.572 (3) Å] and wider angle compared to that facing the side of the mol­ecules with two hydrogen atoms [1.561 (4) Å]. As expected, the axial S—F bond is the longest [1.582 (5) Å]. In the crystal, in-plane C—H⋯F and N⋯I inter­actions as well as out-of-plane F⋯C inter­actions are observed. According to the Hirshfeld analysis, the principal inter­molecular contacts for the title compound are F⋯H (29.4%), F⋯I (15.8%), F⋯N (11.4%), F⋯F (6.0%), N⋯I (5.6%) and F⋯C (4.5%).

## Abstract 2

A novel tris­ubstituted arene compound bearing a penta­fluoro­sulfanyl group (SF_5_) has been synthesized through a regioselective ortho li­thia­tion, improved by the addition of a bidentate amine, followed by trapping with electrophilic I_2_. Incorporating the iodo group in 2-iodo-4-(penta­fluoro-λ^6^-sulfan­yl)benzo­nitrile strengthens the inter­molecular inter­action network with the cyano and penta­fluoro substituents.

## Chemical context   

Organic compounds containing the tri­fluoro­methyl (CF_3_) or penta­fluoro­thio (or penta­fluoro-λ^6^-sulfanyl, SF_5_) groups play an important role in organofluorine chemistry because of their special properties including low surface energy, hydro­phobicity, high chemical resistance, high thermal stability and high electronegativity (Kirsch *et al.*, 1999 ▸, 2014 ▸; Iida *et al.*, 2015 ▸; Beier *et al.*, 2011 ▸). SF_5_, coined as the ‘super-tri­fluoro­meth­yl’ group, is often preferred to CF_3_ as it is more electronegative, lipophilic and chemically stable, and possesses a higher steric effect (Bowden *et al.*, 2000 ▸). The current inter­est in the field of drug discovery of fluorinated substituents is based on the possibility of improving both the metabolic stability and bioavailability of receptor binders upon the incorporation of susbtituents with one or more fluorine atoms (Altomonte *et al.*, 2014 ▸; Savoie & Welch, 2015 ▸; Sowaileh *et al.*, 2017 ▸). In fact, several blockbuster drugs include such a group, demonstrating the prominent role of the tri­fluoro­methyl group in the area of drug discovery (O’Hagan, 2010 ▸; Müller *et al.*, 2007 ▸; Purser *et al.*, 2008 ▸). New mol­ecules incorporating the SF_5_ group are thus potential alternatives to already existing biologically active mol­ecules containing the CF_3_ substitution. Additionally, the chemical robustness of SF_5_ has been explored in other areas such as polymer chemistry (Zhou *et al.*, 2016 ▸). Despite the popularity of the title compound, an important precursor in organofluorine chemistry, its crystallographic characterization, which is an important milestone in the synthesis of next-generation materials containing this motif, has not been reported. Herein, we describe a variation to the synthetic approach and give details of its simple crystallization through slow evaporation methods, yielding X-ray diffraction-quality single crystals.
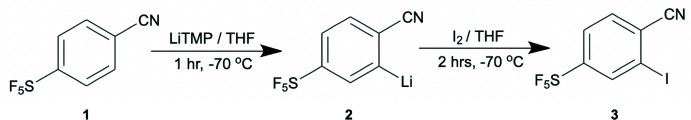


The title compound was obtained as part of our studies toward the synthesis of functionalized arenes containing the SF_5_ moiety. Its synthesis involves a one-pot reaction in which the inter­action of the cyano group in 4-(penta­fluoro­sulfan­yl)benzo­nitrile to the Lewis acidic lithium cation in lithium tetra­methyl­piperidide (LiTMP) allows deprotonation from the nearest *ortho*-H atom on the arene. The SF_5_-containing organolithium species is then quenched with iodine to yield the title compound. This reaction pathway was proposed by Iida *et al.* (2015 ▸) for the synthesis of SF_5_-substituted zinc phthalocyanines. We modified the synthesis by adding tetra­methyl­ethylenedi­amine (TMEDA), an amine additive that serves to break up the li­thia­ted base aggregates, allowing for accelerated reactivity because of the increased basicity. This variation improves the total yield of the title compound by 8%.

## Structural commentary   

**Figure 1:**
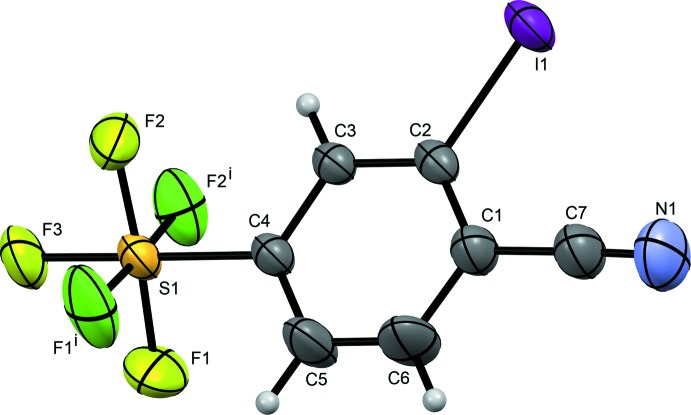
Mol­ecular structure of the title compound, including atom labelling. Displacement ellipsoids are drawn at the 50% probability level. Atoms generated by the mirror plane [symmetry code: (i) *x*, −*y* + 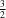, *z*] are depicted in dark green.

Fig. 1 ▸ shows the mol­ecular structure of the title compound, which crystallizes in the space group *Pnma.* Its asymmetric unit comprises a single mol­ecule lying on a mirror plane perpendicular to [010] with the iodo, cyano, and the sulfur and axial fluorine atoms of the penta­fluoro­sulfanyl substituent in the plane of the mol­ecule. The fluorine atoms of the penta­fluoro­sulfanyl group in the equatorial positions lie above and below the plane in a staggered fashion relative to the two hydrogen atoms *ortho* to the substituent; of those, two of the four fluorine atoms are generated symmetrically by the mirror plane. The S1—F_(eq)_ bond distances differ from each other depending on which side of the mol­ecule the bond is located (Table 1 ▸). The S1—F2_(eq)_ bond and its symmetry equivalent S1—F2 ^i^_(eq)_ [symmetry code: (i) *x*, −*y* + 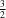, *z*] are on the same side as the iodine atom and exhibit a longer bond distance of 1.572 (3) Å in comparison to S1—F1_(eq)_ and S1—F1^i^_(eq)_, which are further away from the iodine and have a shorter bond length distance of 1.561 (4) Å. The S1—F3_(ax)_ bond length of 1.582 (5) Å is the longest and is consistent with those in similar structures [1.588 (2) and 1.573 (3) Å; Du *et al.*, 2016 ▸].

## Supra­molecular features   

**Figure 2:**
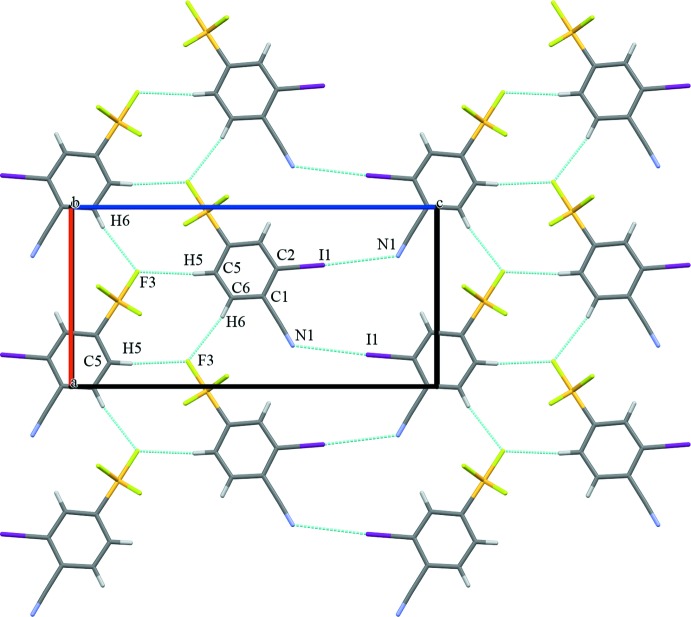
In plane contacts. A view along the *b* axis of crystal packing of the title compound, with short-contact inter­actions shown as dashed lines.

**Figure 3:**
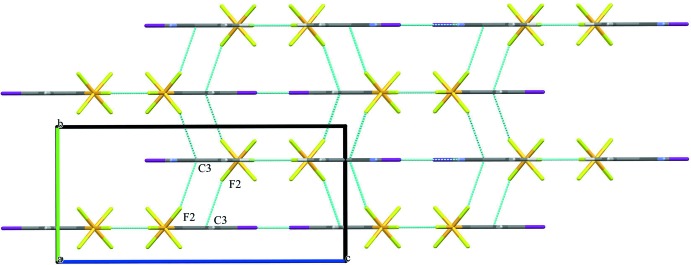
Out-of-plane contacts. Partial packing diagram for the the title compound viewed along the *a* axis. F⋯π inter­actions are shown as dashed lines.

The packing of the title compound is consolidated through a series of inter­molecular inter­actions, which can be classified as being in-plane and out-of-plane (Table 2 ▸). Each mol­ecule acts as a C—H donor through the *meta*- and *para*-hydrogen atoms of the phenyl ring counter to the iodine atom. Two C–H⋯F hydrogen bonds, C5—H5⋯F3 and C6—H6⋯F3 with H⋯F distances of 2.5 and 2.6 Å, respectively, create an in-plane network (Table 2 ▸ and Fig. 2 ▸). Both the H5 and H6 atoms are highly acidic because of the electron-withdrawing effects of the –SF_5_ and –CN substituents. Additionally, significant in-plane halogen-bonding inter­actions [N1⋯I1(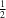 + *x*, 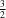 − *y*, 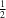 − *z*) = 3.408 (10) Å] are observed (Metrangolo *et al.*, 2005 ▸). Out-of-plane inter­molecular inter­actions arise primarily from F⋯π ring inter­actions at one of the ‘corners’ of the ring (Fig. 3 ▸) with an F2⋯C3(2 − *x*, −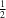 + *y*, 1 − *z*) distance of 3.124 (5) Å.

## Hirshfeld surface analysis   

**Figure 4:**
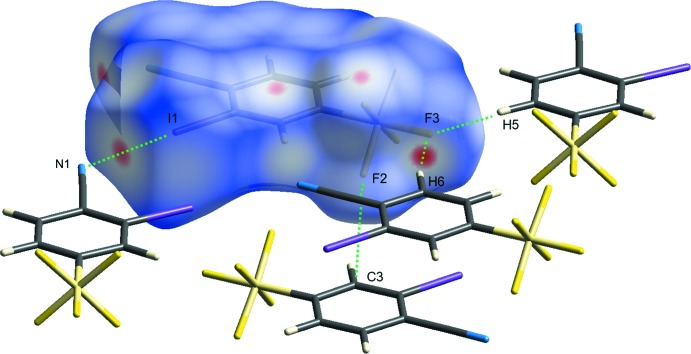
A view of the Hirshfeld surface of the title compound mapped over *d*_norm_ with the four main inter­molecular contacts in the crystal lattice.

**Figure 5:**
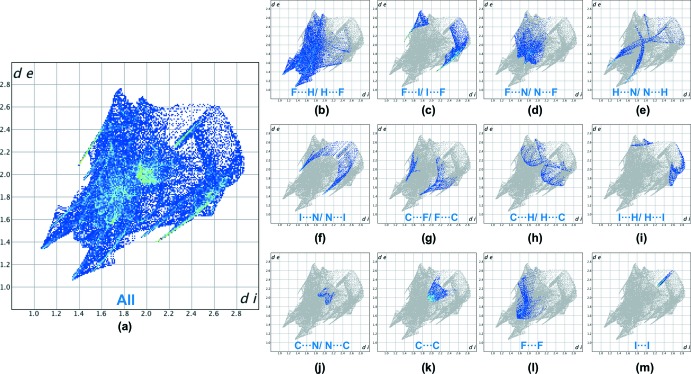
Full (*a*) and individual (*b*)–(*m*) two-dimensional fingerprint plots showing the 12 inter­molecular contacts present in the crystal structure.

The Hirshfeld surface (Spackman & Jayatilaka, 2009 ▸) for the title compound mapped over *d*_norm_ is shown in Fig. 4 ▸ while Fig. 5 ▸ shows the associated two-dimensional fingerprint plots (McKinnon *et al.*, 2007 ▸), both generated with *CrystalExplorer17* (Turner *et al.*, 2017 ▸). Red spots on the Hirshfeld surface mapped over *d*_norm_ in the colour range −0.4869 to 1.4157 arbitrary units confirm the previously mentioned main inter­molecular contacts. The fingerprint plots are given for all contacts and those delineated into F⋯H/H ⋯F (29.4%; Fig. 5 ▸ *b*), F⋯I/I⋯F (15.8%; Fig. 5 ▸ *c*), F⋯N/N⋯F (11.4%; Fig. 5 ▸ *d*), H⋯N/N⋯H (6.3%; Fig. 5 ▸ *e*), I⋯N/N⋯I (5.6%; Fig. 5 ▸ *f*), C⋯F/F⋯C (4.5%; Fig. 5 ▸ *g*), C⋯H/H⋯C (4.5%; Fig. 5 ▸ *h*), I⋯H/H⋯I (3.3%; Fig. 5 ▸ *i*), C⋯N/N⋯C (1.6%; Fig. 5 ▸ *j*), C⋯C (9.5%; Fig. 5 ▸ *k*), F⋯F (6.0%; Fig. 5 ▸ *l*) and I⋯I (2.2%; Fig. 5 ▸ *m*) inter­actions. Thus, the Hirshfeld surface analysis indicates that the most significant contributions arise from F⋯H and F⋯I contacts.

## Database survey   

A search of the Cambridge Structural Database (Version 5.39, updated May 2017; Groom *et al.*, 2016 ▸) revealed no matching compounds with the title compound substructure and the three substituents. However, a search for SF_5_ aryl compounds fragment revealed about 85 hits: 77 of these structures were reported in the last 10 years, which shows the increasing inter­est in the SF_5_ group. Most of these compounds are used as reagents in the synthesis and modification of pharmaceuticals, such as the anti­malarial agent mefloquine (Wipf *et al.*, 2009 ▸) and the anti-obesity drug fenfluramine (Welch *et al.*, 2007 ▸).

## Synthesis and crystallization   

All solvents and reagents were purified prior to being used. 4-(Penta­fluoro­sulfan­yl)benzo­nitrile was obtained commercially and used without further purification. A solution of 2.5 *M n*-butyl lithium in hexa­nes was used. Column chroma­tography was carried out on a column packed with silica gel 70–230 mesh.

The synthesis of the title compound was performed through the regioselective ortho-li­thia­tion of 4-(penta­fluoro­sulfan­yl)benzo­nitrile with lithium tetra­methyl­piperidide (LiTMP) in THF as solvent, favouring the formation of the *ortho* product (1,2,4-substituted arene) over the *meta* product (1,3,4-substituted arene). The *ortho-*metalated product was subsequently quenched with I_2_ to afford the iodinated tris­ubstituted arene. A dry 50 mL Schlenk tube was charged with 4 mL of dry THF and 300 µL of 2,2,6,6-tetra­methyl piperidine (1.75 mmol, 2 eq.) and 262 µL of *N*,*N*,*N*,*N*-tetra­methyl­ethylendi­amine (1.75 mmol) were added under an inert atmosphere. The solution was cooled to 273 K and 700 µL of 2.5 *M n*-butyl lithium in hexane (1.75 mmol, 2 eq.) were added slowly. The reaction mixture was stirred at 273 K for 30 minutes and then cooled to 195 K. A solution containing 200 mg of 4-(penta­fluoro­sulfan­yl)benzo­nitrile (0.872 mmol, 1 eq.) in 4 mL THF was added dropwise: the solution changed from pale yellow to dark brown upon formation of the metalated inter­mediary. After stirring for 1 h at 195 K, a solution of 244 mg I_2_ (0.960 mmol, 1.2 eq.) in 4 mL THF was added dropwise and stirred for 2 h. The mixture was then warmed to room temperature and stirred for 1 h.

The reaction was quenched with water and THF was removed under reduced pressure, followed by extraction with diethyl ether. The combined organic phase was washed with aqueous 0.1 *M* HCl, 0.1 *M* Na_2_S_2_O_3_ and brine, then dried over MgSO_4_. The crude product was purified by column chromatography (9:1, hexa­ne:ethyl acetate) to yield 71 mg (46%) of the pure arene product as a yellow solid (m.p. 367–369 K). Block-like yellow crystals suitable for X-ray diffraction were obtained by slow evaporation of a saturated CH_2_Cl_2_ solution of the 2-iodo-4-(penta­fluoro-λ^6^-sulfan­yl)benzo­nitrile at room temperature over a period of four days. NMR analyses were performed on a Bruker AV-500 spectrometer using chloro­form-*d* as solvent (CDCl_3_). The solvent signals at 7.26 and 77.00 ppm were used as inter­nal standards for proton and carbon, respectively. ^1^H NMR (500 MHz, Chloro­form-*d*) δ 8.31 (*d*, *J* = 2.1 Hz, 1H), 7.89 (*dd*, *J* = 8.6, 2.1 Hz, 1H), 7.75 (*d*, *J* = 8.6 Hz, 1H). ^13^C NMR (125 MHz, CDCl_3_) δ, 98.22, 117.83, 124.10, 126.16, 134.39, 136.82, 156.15.

## Refinement   

Data collection, crystal data and structure refinement parameters are summarized in Table 3 ▸. H atoms were included in geometrically calculated positions and refined as riding atoms with C—H = 0.93 Å and *U*_iso_(H) = 1.2*U*_eq_(C).

## Supplementary Material

Crystal structure: contains datablock(s) I. DOI: 10.1107/S2056989020000365/dx2019sup1.cif

Structure factors: contains datablock(s) I. DOI: 10.1107/S2056989020000365/dx2019Isup4.hkl

Supporting information file. DOI: 10.1107/S2056989020000365/dx2019Isup3.cml

CCDC reference: 1943767

Additional supporting information:  crystallographic information; 3D view; checkCIF report

## Figures and Tables

**Table 1:** Selected bond lengths and angles

S1—F1_(eq)_ and S1—–F1^i^_(eq)_	1.561 (4)
S1—F2_(eq)_ and S1—F2^i^_(eq)_	1.572 (3)
S1—F3_(ax)_	1.582 (5)
	
C4—S1—F2_(eq)_	92.0 (2)
C4—S1—F1_(eq)_	92.2 (2)

Symmetry code: (i) *x*, −*y* + 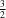, *z*.

**Table 2:** Hydrogen-bond geometry (Å, °)

*D*—H⋯*A*	*D*—H	H⋯*A*	*D*⋯*A*	*D*—H⋯*A*
C5—H5⋯F3^i^	0.93	2.57	3.501 (1)	174
C6—H6⋯F3^ii^	0.93	2.56	3.476 (1)	169

Symmetry codes: (i) 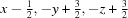; (ii) 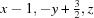.

**Table 3:** Experimental details

Crystal data	
Chemical formula	C_7_H_3_F_5_INS
*M*_r_	355.06
Crystal system, space group	Orthorhombic, *P* *n* *m* *a*
Temperature (K)	300
*a*, *b*, *c* (Å)	8.0634 (1), 7.7088 (1), 16.4410 (3)
*V* (Å^3^)	1021.96 (3)
*Z*	4
Radiation type	Cu *K*α
μ (mm^−1^)	26.99
Crystal size (mm)	0.26 × 0.17 × 0.12

Data collection	
Diffractometer	SuperNova, Single source at offset/far, HyPix3000
Absorption correction	Multi-scan (*CrysAlis PRO*; Rigaku OD, 2018 ▸)
*T*_min_, *T*_max_	0.287, 1.000
No. of measured, independent and observed [*I* > 2σ(*I*)] reflections	9403, 1020, 953
*R*_int_	0.082
(sin θ/λ)_max_ (Å^−1^)	0.605

Refinement	
*R*[*F* ^2^ > 2σ(*F* ^2^)], *wR*(*F* ^2^), *S*	0.042, 0.111, 1.03
No. of reflections	1020
No. of parameters	85
H-atom treatment	H-atom parameters constrained
Δρ_max_, Δρ_min_ (e Å^−3^)	0.74, −1.78

Computer programs: *CrysAlis PRO* (Rigaku OD, 2018 ▸), *SHELXT* (Sheldrick, 2015*a* ▸), *SHELXL* (Sheldrick, 2015*b* ▸) and *OLEX2* (Dolomanov *et al.*, 2009 ▸).

## supplementary crystallographic information

### Crystal data

**Table:**

C_7_H_3_F_5_INS	*D*_x_ = 2.308 Mg m^−^^3^
*M**_r_* = 355.06	Cu *K*α radiation, λ = 1.54184 Å
Orthorhombic, *P**n**m**a*	Cell parameters from 6922 reflections
*a* = 8.0634 (1) Å	θ = 2.7–68.4°
*b* = 7.7088 (1) Å	µ = 26.99 mm^−^^1^
*c* = 16.4410 (3) Å	*T* = 300 K
*V* = 1021.96 (3) Å^3^	Irregular, clear light yellow
*Z* = 4	0.26 × 0.17 × 0.12 mm
*F*(000) = 664	

### Data collection

**Table:**

SuperNova, Single source at offset/far, HyPix3000 diffractometer	953 reflections with *I* > 2σ(*I*)
ω scans	*R*_int_ = 0.082
Absorption correction: multi-scan (CrysAlisPro; Rigaku OD, 2018)	θ_max_ = 68.8°, θ_min_ = 5.4°
*T*_min_ = 0.287, *T*_max_ = 1.000	*h* = −9→9
9403 measured reflections	*k* = −9→9
1020 independent reflections	*l* = −19→19

### Refinement

**Table:**

Refinement on *F*^2^	0 restraints
Least-squares matrix: full	Hydrogen site location: inferred from neighbouring sites
*R*[*F*^2^ > 2σ(*F*^2^)] = 0.042	H-atom parameters constrained
*wR*(*F*^2^) = 0.111	*w* = 1/[σ^2^(*F*_o_^2^) + (0.071*P*)^2^ + 1.8371*P*] where *P* = (*F*_o_^2^ + 2*F*_c_^2^)/3
*S* = 1.03	(Δ/σ)_max_ < 0.001
1020 reflections	Δρ_max_ = 0.74 e Å^−^^3^
85 parameters	Δρ_min_ = −1.77 e Å^−^^3^

### Special details

**Table:**

Geometry. All esds (except the esd in the dihedral angle between two l.s. planes) are estimated using the full covariance matrix. The cell esds are taken into account individually in the estimation of esds in distances, angles and torsion angles; correlations between esds in cell parameters are only used when they are defined by crystal symmetry. An approximate (isotropic) treatment of cell esds is used for estimating esds involving l.s. planes.
Refinement. ShelXL

### Fractional atomic coordinates and isotropic or equivalent isotropic displacement parameters (Å^2^)

**Table:**

	*x*	*y*	*z*	*U*_iso_*/*U*_eq_	
I1	0.67725 (6)	0.750000	0.31142 (3)	0.0540 (3)	
S1	0.97572 (19)	0.750000	0.62917 (9)	0.0398 (4)	
F3	1.1407 (6)	0.750000	0.6812 (3)	0.0641 (14)	
F2	1.0576 (4)	0.6065 (5)	0.5743 (2)	0.0641 (9)	
F1	0.9063 (5)	0.8925 (6)	0.68738 (19)	0.0819 (13)	
C4	0.7869 (8)	0.750000	0.5682 (4)	0.0356 (13)	
C3	0.8020 (7)	0.750000	0.4841 (4)	0.0335 (13)	
H3	0.905856	0.750000	0.459508	0.040*	
C2	0.6572 (8)	0.750000	0.4373 (4)	0.0352 (13)	
C1	0.5034 (8)	0.750000	0.4752 (4)	0.0467 (16)	
C7	0.3514 (9)	0.750000	0.4290 (5)	0.056 (2)	
C5	0.6348 (10)	0.750000	0.6059 (5)	0.065 (3)	
H5	0.628167	0.750000	0.662401	0.078*	
N1	0.2295 (10)	0.750000	0.3943 (6)	0.080 (2)	
C6	0.4948 (10)	0.750000	0.5606 (5)	0.072 (3)	
H6	0.391926	0.750000	0.586180	0.087*	

### Atomic displacement parameters (Å^2^)

**Table:**

	*U*^11^	*U*^22^	*U*^33^	*U*^12^	*U*^13^	*U*^23^
I1	0.0575 (4)	0.0786 (4)	0.0259 (3)	0.000	−0.00566 (16)	0.000
S1	0.0409 (8)	0.0534 (9)	0.0250 (8)	0.000	−0.0044 (6)	0.000
F3	0.055 (3)	0.097 (4)	0.040 (3)	0.000	−0.020 (2)	0.000
F2	0.0622 (18)	0.0697 (19)	0.0605 (18)	0.0256 (16)	−0.0188 (15)	−0.0192 (17)
F1	0.081 (3)	0.110 (3)	0.055 (2)	0.022 (2)	−0.0146 (16)	−0.044 (2)
C4	0.038 (3)	0.045 (3)	0.024 (3)	0.000	−0.002 (3)	0.000
C3	0.037 (3)	0.038 (3)	0.026 (3)	0.000	0.000 (2)	0.000
C2	0.047 (4)	0.033 (3)	0.025 (3)	0.000	−0.003 (2)	0.000
C1	0.038 (3)	0.066 (4)	0.036 (4)	0.000	−0.003 (3)	0.000
C7	0.046 (4)	0.081 (6)	0.042 (5)	0.000	−0.004 (3)	0.000
C5	0.047 (4)	0.121 (8)	0.027 (4)	0.000	0.002 (3)	0.000
N1	0.049 (4)	0.121 (7)	0.071 (5)	0.000	−0.016 (4)	0.000
C6	0.036 (4)	0.139 (9)	0.042 (4)	0.000	0.014 (3)	0.000

### Geometric parameters (Å, º)

**Table:**

I1—C2	2.076 (6)	C3—H3	0.9300
S1—F3	1.582 (5)	C3—C2	1.399 (8)
S1—F2	1.572 (3)	C2—C1	1.388 (9)
S1—F2^i^	1.572 (3)	C1—C7	1.442 (10)
S1—F1^i^	1.561 (4)	C1—C6	1.406 (11)
S1—F1	1.561 (4)	C7—N1	1.136 (10)
S1—C4	1.823 (7)	C5—H5	0.9300
C4—C3	1.388 (9)	C5—C6	1.353 (11)
C4—C5	1.374 (10)	C6—H6	0.9300
			
F3—S1—C4	179.4 (3)	C5—C4—C3	121.8 (6)
F2^i^—S1—F3	87.52 (18)	C4—C3—H3	120.8
F2—S1—F3	87.52 (18)	C4—C3—C2	118.4 (6)
F2^i^—S1—F2	89.4 (3)	C2—C3—H3	120.8
F2—S1—C4	92.04 (19)	C3—C2—I1	118.9 (5)
F2^i^—S1—C4	92.04 (19)	C1—C2—I1	121.2 (5)
F1^i^—S1—F3	88.3 (2)	C1—C2—C3	119.9 (6)
F1—S1—F3	88.3 (2)	C2—C1—C7	121.6 (6)
F1—S1—F2^i^	90.4 (2)	C2—C1—C6	119.5 (6)
F1—S1—F2	175.8 (2)	C6—C1—C7	118.9 (7)
F1^i^—S1—F2	90.4 (2)	N1—C7—C1	178.4 (9)
F1^i^—S1—F2^i^	175.8 (2)	C4—C5—H5	120.1
F1^i^—S1—F1	89.5 (4)	C6—C5—C4	119.8 (7)
F1^i^—S1—C4	92.2 (2)	C6—C5—H5	120.1
F1—S1—C4	92.2 (2)	C1—C6—H6	119.7
C3—C4—S1	118.3 (5)	C5—C6—C1	120.6 (7)
C5—C4—S1	119.8 (5)	C5—C6—H6	119.7
			
I1—C2—C1—C7	0.000 (2)	F1^i^—S1—C4—C5	44.79 (18)
I1—C2—C1—C6	180.000 (2)	C4—C3—C2—I1	180.000 (1)
S1—C4—C3—C2	180.000 (2)	C4—C3—C2—C1	0.000 (2)
S1—C4—C5—C6	180.000 (2)	C4—C5—C6—C1	0.000 (3)
F2—S1—C4—C3	−44.74 (15)	C3—C4—C5—C6	0.000 (3)
F2^i^—S1—C4—C3	44.74 (15)	C3—C2—C1—C7	180.000 (2)
F2^i^—S1—C4—C5	−135.26 (15)	C3—C2—C1—C6	0.000 (2)
F2—S1—C4—C5	135.26 (15)	C2—C1—C6—C5	0.000 (3)
F1^i^—S1—C4—C3	−135.21 (18)	C7—C1—C6—C5	180.000 (2)
F1—S1—C4—C3	135.21 (18)	C5—C4—C3—C2	0.000 (2)
F1—S1—C4—C5	−44.79 (18)		

Symmetry code: (i) *x*, −*y*+3/2, *z*.

### Hydrogen-bond geometry (Å, º)

**Table:**

*D*—H···*A*	*D*—H	H···*A*	*D*···*A*	*D*—H···*A*
C5—H5···F3^ii^	0.93	2.57	3.501 (1)	174
C6—H6···F3^iii^	0.93	2.56	3.476 (1)	169

Symmetry codes: (ii) *x*−1/2, −*y*+3/2, −*z*+3/2; (iii) *x*−1, −*y*+3/2, *z*.

### Non-covalent intermolecular interactions (Å)

**Table:**

N1—I1	3.408	F2—C3'	3.408
F3—H5	3.123	F3—H6	2.573, 2.558

### Hydrogen-bond and short-contact geometry (Å, °)

**Table:**

*D*—H···*A*/*D*···*A*	*D*—H	H···*A*	*D*···*A*	*D*—H···*A*
C5—H5···F3	0.93	2.57	3.501 (1)	174
F2···C3	–	–	3.123 (1)	–
C6—H6···F3	0.93	2.56	3.476 (1)	169
N1···I1	–	–	3.408 (1)	-

### Percentage contributions of interatomic contacts to the Hirshfeld surface

**Table:**

Contact	% contribution	Contact	% contribution
F···H/H···F	29.4	C···C	9.5
F···I/I···F	15.8	F···F	6.0
F···N/N···F	11.4	I···I	2.2
H···N/N···H	6.3		
I···N/N···I	5.6		
C···F/F···C	4.5		
C···H/H···C	4.5		
I···H/H···I	3.3		
C···N/N···C	1.6		

## References

[bb1] Altomonte, S., Baillie, G. L., Ross, R. A., Riley, J. & Zanda, M. (2014). *RSC Adv.* **4**, 20164–20176.

[bb2] Beier, P., Pastýříková, T. & Iakobson, G. (2011). *J. Org. Chem.* **76**, 4781–4786. 10.1021/jo200618p 21545179

[bb3] Bowden, R. D., Comina, P. J., Greenhall, M. P., Kariuki, B. M., Loveday, A. & Philp, D. (2000). *Tetrahedron*, **56**, 3399–3408.

[bb4] Dolomanov, O. V., Bourhis, L. J., Gildea, R. J., Howard, J. A. K. & Puschmann, H. (2009). *J. Appl. Cryst.* **42**, 339–341.

[bb5] Du, J., Hua, G., Beier, P., Slawin, A. M. Z. & Woollins, J. D. (2016). *Struct. Chem*. 28, 723–733.

[bb6] Groom, C. R., Bruno, I. J., Lightfoot, M. P. & Ward, S. C. (2016). *Acta Cryst.* B**72**, 171–179. 10.1107/S2052520616003954 PMC4822653 27048719

[bb7] Iida, N., Tanaka, K., Tokunaga, E., Mori, S., Saito, N. & Shibata, N. (2015). *Chem. Open.* **4**, 698–702. 10.1002/open.201500165 PMC4906510 27308194

[bb8] Kirsch, P. & Bremer, M. (2014). *Chimia*, **68**, 363–370. 10.2533/chimia.2014.363 25198746

[bb9] Kirsch, P., Bremer, M., Heckmeier, M. & Tarumi, K. (1999). *Angew. Chem. Int. Ed.* **38**, 1989–1992. 10.1002/(SICI)1521-3773(19990712)38:13/14<1989::AID-ANIE1989>3.0.CO;2-K 34182706

[bb10] McKinnon, J. J., Jayatilaka, D. & Spackman, M. A. (2007). *Chem. Commun.* pp. 3814–3816. 10.1039/b704980c 18217656

[bb11] Metrangolo, P., Neukirch, H., Pilati, T. & Resnati, G. (2005). *Acc. Chem. Res.* **38**, 386–395. 10.1021/ar0400995 15895976

[bb12] Müller, K., Faeh, C. & Diederich, F. (2007). *Science*, **317**, 1881–1886. 10.1126/science.1131943 17901324

[bb13] O’Hagan, D. (2010). *J. Fluor. Chem.* **131**, 1071–1081.

[bb14] Purser, S., Moore, P. R., Swallow, S. & Gouverneur, V. (2008). *Chem. Soc. Rev.* **37**, 320–330. 10.1039/b610213c 18197348

[bb23] Rigaku OD (2018). *CrysAlis PRO*. Rigaku Oxford Diffraction, Yarnton, England.

[bb15] Savoie, P. R. & Welch, J. T. (2015). *Chem. Rev.* **115**, 1130–1190. 10.1021/cr500336u 25341449

[bb16] Sheldrick, G. M. (2015*a*). *Acta Cryst.* A**71**, 3–8.

[bb24] Sheldrick, G. M. (2015*b*). *Acta Cryst.* C**71**, 3–8.

[bb17] Sowaileh, M. F., Hazlitt, R. A. & Colby, D. A. (2017). *Med. Chem*. 12, 1481–1490. 10.1002/cmdc.201700356 28782186

[bb18] Spackman, M. A. & Jayatilaka, D. (2009). *CrystEngComm*, **11**, 19–32.

[bb19] Turner, M. J., McKinnon, J. J., Wolff, S. K., Grimwood, D. J., Spackman, P. R., Jayatilaka, D. & Spackman, M. A. (2017). *CrystalExplorer17*. University of Western Australia. http://hirshfeldsurface. net

[bb20] Welch, J. T. & Lim, D. S. (2007). *Bioorg. Med. Chem.* **15**, 6659–6666. 10.1016/j.bmc.2007.08.012 17765553

[bb21] Wipf, P., Mo, T., Geib, S. J., Caridha, D., Dow, G. S., Gerena, L., Roncal, N. & Milner, E. E. (2009). *Org. Biomol. Chem.* **7**, 4163–4165. 10.1039/b911483a PMC2929370 19795052

[bb22] Zhou, Y., Wang, J., Gu, Z., Wang, S., Zhu, W., Aceña, J. L., Soloshonok, V. A., Izawa, K. & Liu, H. (2016). *Chem. Rev.* **116**, 422–518. 10.1021/acs.chemrev.5b00392 26756377

